# The EQ-5D-5L valuation study for Trinidad and Tobago

**DOI:** 10.1186/s12955-024-02266-7

**Published:** 2024-07-02

**Authors:** Henry Bailey, Marcel F. Jonker, Eleanor Pullenayegum, Fanni Rencz, Bram Roudijk

**Affiliations:** 1 Department of Economics, The University of the West Indies, St Augustine Campus, St Augustine, Trinidad and Tobago; 2 HEU, Centre for Health Economics, The University of the West Indies, St Augustine Campus, St Augustine, Trinidad and Tobago; 3 Erasmus School of Health Policy & Management, Erasmus University Rotterdam, Rotterdam, the Netherlands; 4 Erasmus Centre for Health Economics Rotterdam, Erasmus University Rotterdam, Rotterdam, The Netherlands; 5 Erasmus Choice Modelling Centre, Erasmus University Rotterdam, Rotterdam, The Netherlands; 6 Child Health Evaluative Sciences, The Hospital for Sick Children; Dalla Lana School of Public Health, University of Toronto, Toronto, Canada; 7 Department of Health Policy, Corvinus University of Budapest, Budapest, Hungary; 8 EuroQol Research Foundation, Rotterdam, the Netherlands; 9 Department of Psychiatry, Erasmus University Medical Center, Rotterdam, the Netherlands

**Keywords:** EQ-5D-5L, Trinidad and Tobago, EQ-VT, QALY

## Abstract

**Purpose:**

The 2016 EQ-5D-3L value set for Trinidad and Tobago (T&T) allows for the calculation of EQ-5D-5L values via the crosswalk algorithm. The 2016 value set was based on methods predating the EQ-VT protocol, now considered the gold standard for developing EQ-5D value sets. Furthermore, direct elicitation of EQ-5D-5L is preferred over crosswalked values. This study aimed to produce an EQ-5D-5L value set for T&T.

**Methods:**

A representative sample (age, sex, geography) of adults each completed 10 composite Time Trade-Off (cTTO) tasks and 12 Discrete Choice Experiment (DCE) tasks in face-to-face interviews. The cTTO data were analyzed using a Tobit model that corrects for heteroskedasticity. DCE data were analyzed using a mixed logit model. The cTTO and DCE data were combined in hybrid models.

**Results:**

One thousand and seventy-nine adults completed the valuation interviews. Among the modelling approaches that were explored, the hybrid heteroskedastic Tobit model produced all internally consistent, statistically significant coefficients, and performed best in terms of out-of-sample predictivity for single states. Compared to the existing EQ-5D-5L crosswalk set, the new value set had a higher number of negative values (236 or 7.6% versus 21 or 0.7%). The mean absolute difference was 0.157 and the correlation coefficient between the two sets was 0.879.

**Conclusion:**

This study provides a value set for the EQ-5D-5L for T&T using the EQ-VT protocol. We recommend this value set for QALY computations relating to T&T.

## Introduction

The EQ-5D-5L instrument is a health classification system that is used for health outcomes measurement in developed, and now increasingly also in developing countries [1]. EQ-5D-5L has many uses in healthcare and health economics including its use as the adjustment for Quality Adjusted Life Years (QALYs), for cost utility analysis and in quantifying burden of illness. EQ-5D-5L comprises 5 dimensions in order: mobility, self-care, usual activities, pain/discomfort and anxiety/depression, on each of which a respondent can indicate problems at one of 5 levels: no, slight, moderate, severe or extreme problems/unable to. With 5 dimensions in 5 levels, there are 5^5^ = 3,125 possible states of health that can be described using the classification system. Each of these health states can be presented as a five-digit string. For example, if a respondent indicates he/she has moderate problems (level 3) with walking about, slight problems (level 2) with self care, no problems (level 1) with usual activities, severe (level 4) pain/discomfort and is extremely (level 5) anxious or depressed, the health state can be coded as state “32145”, representing the level of problems on each dimension, in order of appearance. In an EQ-5D-5L valuation study, a societal value is obtained for each EQ-5D-5L state relative to all other EQ-5D-5L states [2]. This is known as the index value for the state and the collection of all 3,125 index values for a population is known as a value set. Different countries will have different EQ-5D-5L value sets as the preferences among states of health are known to be driven by many factors, including factors relating to national culture [3].

The original EQ-5D instrument (EQ-5D-3L) had 3 levels [4]. This was similar to EQ-5D-5L but without the two intermediate levels: slight and severe and using ‘confined to bed’ instead of ‘unable to walk about’ for the highest level of problems with mobility. An EQ-5D-3L valuation study was undertaken for Trinidad and Tobago in 2016 [5]. This has been used to produce a set of EQ-5D-3L population norms for Trinidad and Tobago and in several applications [6–8]. EQ-5D-5L was introduced to increase the sensitivity of the instrument [9]. A crosswalk EQ-5D-5L value set was developed for Trinidad and Tobago based on the EQ-5D-3L value set [10]. The Trinidad and Tobago crosswalk value set has been used in several applications in Trinidad and Tobago [11–15] and the Trinidad and Tobago EQ-5D-5L crosswalk value set has also been used in other countries in the Caribbean region [16–18]. While an EQ-5D-5L crosswalk value set is known to be more sensitive than EQ-5D-3L, a directly elicited EQ-5D-5L value set is preferred over a crosswalk. Directly elicited value sets are not subject to the assumptions underlying mapped value sets, for example the Van Hout et al. crosswalk algorithm was based mostly on responses from European respondents which may not necessarily be representative for countries with different cultures [10].

Given the growing role that EQ-5D health outcomes plays in clinical practice and disease studies, and the potential that it offers for policy work in Trinidad and Tobago and the wider Caribbean, a decision was made to develop a directly elicited EQ-5D-5L value set for Trinidad and Tobago. The EuroQol Group has developed and published a standardized protocol for EQ-5D-5L valuation studies known as EQ-VT [19]. This study reports the application of EQ-VT in Trinidad and Tobago. The goal of the study was to develop a value set for the EQ-5D-5L, by directly assessing the preferences for EQ-5D-5L health states in the population of Trinidad and Tobago.

## Methods

This study followed the EQ-VT protocol, version 2.1 [19]. Computer-assisted personal interviews were utilized in which respondents completed Composite Time Trade Off (cTTO) and Discrete Choice Experiment (DCE) tasks. These data were subsequently modelled to derive a national EQ-5D-5L value set for Trinidad and Tobago. This value set was then compared against the existing Trinidad and Tobago EQ-5D-5L crosswalk value set that was based on the EQ-5D-3L value set. We followed the CREATE checklist for reporting Valuation Studies of Multi-Attribute Utility-Based Instruments [20].

### Valuation methods

The cTTO combines the traditional Time Trade Off (TTO) method for health states considered better than dead (BTD), and lead-time TTO (LT-TTO) for states that respondents consider to be worse than dead (WTD) [21]. Both methods follow an iterative procedure in which respondents choose between living in two different hypothetical lives. In the TTO, Life A is described as living for a number of life years in full health, and Life B being 10 years in some EQ-5D-5L health state. Depending on the choice made by the respondent, the number of years in full health in Life A is subsequently varied, until the respondent is indifferent between the two lives, and a value can be inferred for the health state of Life B. The LT-TTO is invoked when respondents indicate that they consider a health state to be WTD, and follows a similar iterative procedure, except that the 10 years in Life B are now preceeded by 10 years in full health. Further details can be found elsewhere [19, 21].

In the DCE task, respondents were asked to choose which of two different EQ-5D-5L health states they prefer, without any duration of time spent in these health states specified. In contrast to the iterative cTTO method, which produces cardinal values, the DCE task encompasses a single choice, which produces binary outcomes, from which no direct value for a health state can be inferred.

### Interview procedures

Respondents were interviewed in computer-assisted personal interviews, following the standardized EQ-VT interview protocol and interviewer script. First, respondents were presented with information about the aims and the content of the study, and completed an informed consent form. Subsequently, respondents completed a warm-up exercise, which included some demographic questions, a self-completion EQ-5D-5L questionnaire and accompanying EuroQol Visual Analogue scale (EQ VAS). After that, the cTTO task was introduced. Respondents were first presented with an example task in which they valued the health state “being in a wheelchair”. Here, the task was explained to the respondents after which they completed the example question. This was followed by another example question: “a health state much better than being in a wheelchair” or “a health state much worse than being in a wheelchair”, depending on whether the respondent considered “being in a wheelchair” as BTD or WTD. This was followed by another 3 practice questions, using EQ-5D-5L health states (states 21121, 35554 and 15411, representing mild, severe and potentially difficult to imagine health states, respectively). Subsequently, respondents valued 10 EQ-5D-5L health states using the cTTO task. After completing the cTTO tasks, respondents were shown the feedback module: a rank order of their answers, after which they could indicate whether any of the responses were in the wrong order [22]. Lastly, respondents were presented with a set of 12 DCE choice pairs, followed by a short demographic survey (with the remaining demographic questions) after which they were thanked for their participation and invited to choose a gift valued at 60 Trinidad and Tobago dollars (about $9 USD) from range of options including gift vouchers, tote bags, water bottles etc. as compensation for their time.

### Selection of health states

For the cTTO task, the standard EQ-VT health state design was used comprising 86 health states distributed over 10 blocks of 10 health states. Each block consisted of one mild health state (one of the following: 21111, 12111, 11211, 11121 and 11112), the worst health state 55555 and a set of 8 states chosen from an efficient design comprising 80 health states [23]. For the DCE tasks, respondents were allocated a block of 12 choice pairs out of 20 unique blocks from a Bayesian efficient design using priors from a set of 19 different EQ-5D-5L valuation studies. The design is described in Appendix C. Each respondent was randomly allocated a block of cTTO states and DCE choice pairs.

### Quality control

The EQ-VT quality control (QC) procedures were implemented to ensure adequate data quality. We followed the Ramos-Goñi et al. protocol in which interviews were flagged as potentially non-compliant to the interview protocol if at least one of the following conditions was met: 1) the interviewer did not explain the WTD part of the cTTO task in at least one of the wheelchair practice tasks, 2) the interviewer spent less than 3 min on the two wheelchair tasks, 3) the respondent completed the main 10 cTTO tasks in less than 5 min, and 4) state 55555 did not receive the lowest value and another state received a value that was at least 0.5 lower [24]. Data were collected in batches of 10 interviews, after which the data were examined, and the number of interviews that were flagged per interviewer. If more than 40% of interviews were flagged during a single round of interviews, the interviewer failed the QC, their batch of data was removed and the interviewer was retrained. If an interviewer failed the QC twice, the interviewer was removed from the study.

### Sampling

A nationally representative sample of 1000 respondents aged 18 years and over was targeted. Quota sampling was performed based on age and sex, as well as the 14 administrative regions of Trinidad and the combined 7 parishes of Tobago based on the 2011 Population and Housing Census for Trinidad and Tobago (Central Statistical Office (CSO) of Trinidad and Tobago) which was the most recent census data available for Trinidad and Tobago. Streets were randomly selected from the CSO maps and 1 in every 4 households were visited on each selected street. One member of each household was selected using the most recent birthday method and invited to take part in the survey. The respondents were recruited by a local market research company. A team of 14 interviewers, employed by the market research company, was trained during a 1-week training session by two members of the research team (HB and BR). Subsequently, each interviewer completed at least two sets of 5 pilot interviews. Four interviewers failed the QC procedures during the pilot phase or thereafter, and were removed from the study. A team of 10 remaining interviewers completed the data collection. Furthermore, interviewer effects were monitored by assessing whether the interviewers produced roughly similar distributions of values. The data were collected between July and September 2022.

### Analyses

Several 20-parameter models, with each parameter representing the difference between having no problems and having a certain level of problems on a particular dimension, were estimated on the data. The cTTO and DCE data were modelled in isolation as well as jointly. Details on the functional form of these models can be found in Appendix B.

We first estimated a random intercept model on the cTTO data to account for the nested structure of the cTTO data (respondents each complete multiple cTTO tasks) and a random left-censored intercept Tobit model (to account for the nesting of the data as well as the fact that respondents cannot assign values lower than -1 to any health state). Furthermore, we estimated models that corrected for the heteroskedastic nature of the cTTO data, with and without the Tobit link to account for the censored nature of the data. The regression constant was suppressed in cases where it was not significant.

The DCE data were analyzed using conditional logit and mixed logit models with the latter accounting for preference heterogeneity. Furthermore, hybrid models were estimated which used a joint likelihood function to model the cTTO and DCE data in combination [25]. Hybrid models were estimated taking into account the heteroskedastic nature of the data, as well as the using the Tobit link for the cTTO data. Each model that corrects for heteroskedasticity in the cTTO data, as well as the hybrid models were estimated without a constant when the constant was not significant. Lastly, sensitivity analyses were carried out by estimating the cTTO and hybrid models while leaving out the responses flagged in the feedback module. A final model was selected based on the properties of the model, such as whether heteroskedasticity was present and whether there was a substantial number of censored responses. Furthermore, model fit and predictivity was considered using mean absolute error (MAE). MAE was calculated over all responses as well as how well the models predicted the mean observed cTTO value for the 86 health states included in the cTTO health state design. Lastly, out-of-sample predictivity was tested using a leave-one-out analysis, in which models were estimated by leaving out a single state, predicting its value and evaluating the MAE of the model. The same procedure was used by leaving out a block of health states rather than a single health state. Data analyses were performed in Stata 18, using the xtreg, xttobit, intreg, clogit, mixlogit and hyreg commands.

## Results

### Demographics

A representative sample (age, sex, geography) of 1,079 adults completed the EQ-VT valuation tasks in face-to-face interviews. The response rate was 34%. Table 1 shows a breakdown of the age and sex distribution of the sample compared with the population over age 18, and Table 2 shows the geographic composition of the sample compared with the population. All comparisons were done against the 2011 census data.

**Table 1:** Age and sex composition of the sample compared with the population

Age group	Sample		Population	
	**Male**	**Female**	**Male**	**Female**
**18–24**	7.7%	7.4%	7.7%	7.7%
**25–34**	10.4%	11.3%	11.6%	11.4%
**35–44**	9.5%	10.9%	9.1%	8.9%
**45–54**	6.3%	8.2%	9.3%	9.1%
**55–64**	6.0%	10.2%	6.7%	6.6%
**65 + **	5.5%	6.7%	5.4%	6.4%
**Total**	45.3%	54.7%	49.8%	50.2%

**Table 2:** The geographic composition of the sample compared with the population

Region	Sample	Population
Arima	1.6%	2.7%
Chaguanas	3.2%	6.5%
Couva / Tabaquite / Talparo	16.6%	12.6%
Diego Martin	8.4%	8.2%
Mayaro / Rio Claro	2.9%	2.6%
Penal / Debe	4.2%	6.5%
Pt Fortin	1.7%	1.8%
Port of Spain	3.9%	3.8%
Princes Town	7.5%	7.1%
San Fernando	5.4%	4.4%
San Juan / Laventille	13.7%	12.2%
Sangre Grande	5.4%	5.0%
Siparia	6.8%	6.3%
Tobago	5.8%	4.8%
Tunapuna / Piarco	13.2%	15.8%
Total	100.0%	100.0%

Ethnicity in the sample generally reflected the 2011 census data for the population aged 18 + with Afro- ethnicity slightly under-represented, and mixed/other slightly over-represented. Table 3 shows that the sample appeared to be more educated than the 2011 census data with 24.8% of the sample being tertiary/university educated versus 11.5%.

**Table 3:** Ethnicity and education of the sample compared with population data (aged 18 +) from the 2011 Population and Housing Census for Trinidad and Tobago

	2022 Sample	2011 Census
Ethnicity		
Afro-	34.2%	37.1%
Indo-	40.6%	40.0%
Mixed/Other	25.2%	22.9%
Education		
Less than complete secondary	18.9%	28.8%
Complete secondary	43.5%	51.5%
Vocational	12.9%	8.2%
Tertiary / University	24.8%	11.5%

### Valuation results

**Fig. 1:**
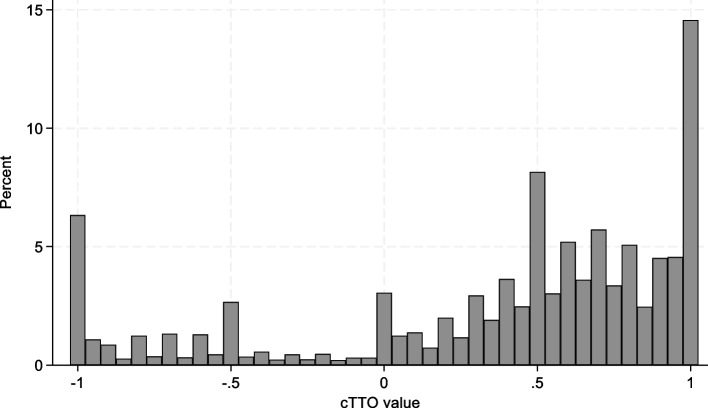
Responses to the cTTO task

Each of the 1079 respondents completed 10 TTO tasks and 12 DCE tasks, giving a total of 10,790 TTO tasks and 12,948 DCE tasks. The average completion time was 43 min and 7 s (standard deviation 18 min and 10 s). In the QC process 164 interviews (15%) were flagged. In all, 63 interviews were flagged for not explaining the WTD task, 18 for inconsistencies with state 55555, 45 for not spending enough time on the wheelchair examples, and 50 for not spending enough time on the cTTO tasks. There were 2 non-traders (assigning the full health value to all states) in the sample. Figure 1 shows the distribution of responses in the cTTO task.

There was some clustering of responses at 1, 0.5 and -1, and 19.27% of responses were negative. There were some differences in the proportion of responses at 1, 0.5 and -1, between interviewers, suggesting that there may have been some minor interviewer effects. The proportion of responses equalling zero was 3.05%, while 6.33% of responses were at -1, indicating the share of potentially censored data. A Breusch-Pagan test showed that there was heteroskedasticity present in the modelled data. Because of the presence of heteroskedasticity and a relatively large share of potentially censored observations at -1, only models correcting for heteroskedasticity and accounting for censoring in the cTTO data were considered, as well as DCE-only models. Table 4 shows the estimated coefficients of selected cTTO-only, DCE-only and hybrid models. Other models are reported in Appendix A, Tables 5, 6 and 7.

**Table 4:** Modelling results of the best performing models for cTTO-only, DCE-only and hybrid

**Heteroskedastic Tobit (cTTO only)**			**Mixed logit (DCE only)**			**Hybrid heteroskedastic Tobit (value set)**	
**coefficients**	**Beta**	**SE**	**Mean Beta**	**SE**	**Beta**^**a**^	**Beta**	**SE**
**mo2**	**0.014**	0.007	0.745**	0.098	**0.048**	**0.027**	0.005
**mo3**	**0.058**	0.013	1.509**	0.123	**0.097**	**0.085**	0.006
**mo4**	**0.162**	0.014	3.165**	0.179	**0.203**	**0.187**	0.006
**mo5**	**0.357**	0.014	5.701**	0.298	**0.365**	**0.368**	0.007
**sc2**	**0.029**	0.007	0.473**	0.11	**0.030**	**0.024**	0.004
**sc3**	**0.074**	0.011	1.236**	0.111	**0.079**	**0.072**	0.006
**sc4**	**0.159**	0.013	2.427**	0.15	**0.156**	**0.150**	0.006
**sc5**	**0.221**	0.012	3.773**	0.197	**0.242**	**0.232**	0.006
**ua2**	**0.016**	0.007	**0.173**	0.108	**0.011**	**0.011**	0.004
**ua3**	**0.087**	0.011	0.961**	0.117	**0.062**	**0.065**	0.006
**ua4**	**0.145**	0.011	2.152**	0.14	**0.138**	**0.146**	0.006
**ua5**	**0.216**	0.013	3.186**	0.176	**0.204**	**0.219**	0.006
**pd2**	**0.026**	0.006	1.259**	0.111	**0.081**	**0.044**	0.004
**pd3**	**0.102**	0.013	2.373**	0.139	**0.152**	**0.128**	0.006
**pd4**	**0.316**	0.013	5.031**	0.238	**0.322**	**0.311**	0.007
**pd5**	**0.541**	0.016	7.730**	0.34	**0.495**	**0.480**	0.008
**ad2**	**0.024**	0.006	0.263*	0.119	**0.017**	**0.020**	0.004
**ad3**	**0.057**	0.012	1.237**	0.137	**0.079**	**0.074**	0.006
**ad4**	**0.168**	0.012	2.436**	0.192	**0.156**	**0.161**	0.006
**ad5**	**0.272**	0.011	3.988**	0.246	**0.256**	**0.264**	0.006
**MAE (total)**	**0.295**		**N/A**		**0.303**	**0.297**	
**MAE(86 states)**	**0.043**		**N/A**		**0.053**	**0.044**	
**MAE out of sample (state)**	**0.056**		**N/A**		**N/A**	**0.049**	
**MAEout of sample (block)**	**0.042**		**N/A**		**N/A**	**0.050**	
**v(55555)**	**-0.607**		**24.38**		**-0.563**	**-0.563**	

Rows mo2-ad5 indicate the decrements associated to specific level-dimension combinations, with for example ua4 representing level 4 problems with usual activities

*AIC* Akaike Information Criterion, *BIC* Bayesian Information Criterion, *MAE* Mean Absolute Error

^a^The estimated mean betas for the mixed logit model were rescaled using the scale length for the hybrid heteroskedastic model. Rescaling factor: 0.0641

*indicates *p*<0.05

**indicates *p*<0.01

In all models pain/discomfort received the highest weight followed by mobility, anxiety/depression, self-care and lastly, usual activities. The value for the worst health state, state 55555, ranged between -0.563 (hybrid heteroskedastic Tobit) and -0.607 (heteroskedastic Tobit model), although other Tobit models, which did not correct for heteroskedasticity, produced lower values (see Appendix A). The difference between the CTTO-only heteroskedastic Tobit model and the hybrid heteroskedastic Tobit model was small in terms of fit statistics such as the MAE. In terms of out-of-sample predictivity, the hybrid heteroskedastic Tobit model performed better than the cTTO-only heteroskedastic Tobit model on removing a single state from the design, while the cTTO-only heteroskedastic Tobit model performed better on removing a whole block. Robustness on out-of-sample predictivity for single states was considered more important, and therefore, the hybrid heteroskedastic Tobit model was selected as the final model, to be used as the value set for Trinidad and Tobago. The value set then takes the following form:

$$\begin{array}{cc}2)&U=1-0.027MO2-0.085MO3-0.187MO4-0.368MO5-0.024SC2-0.072SC3-0.150SC4-0.232SC5-0.011UA2-0.065UA3-0.146UA4-0.219UA5-0.044PD2-0.128PD3-0.311PD4-0.480PD5-0.020AD2-0.074AD3-0.161AD4-0.264AD5\\\end{array}$$

This means that for example health state 21354 would receive the following value: $U\left(21354\right)= 1- 0.027MO2-0.065UA3-0.480PD5-0.161AD4=0.267$.

**Fig. 2:**
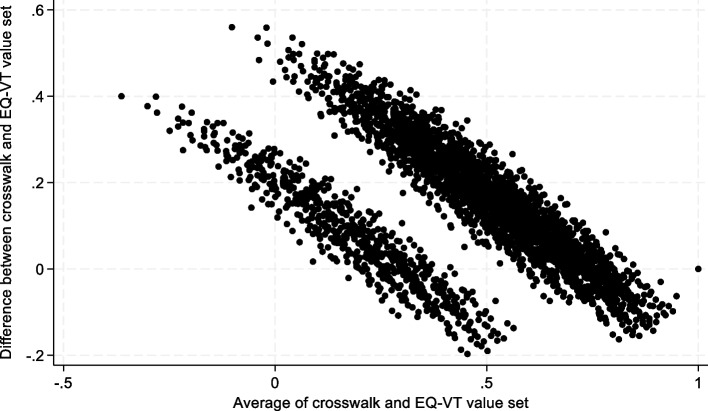
Bland–Altman plot for the crosswalk versus the EQ-VT value set On the vertical axis, the difference between the crosswalk and EQ-VT value sets is shown for all 3125 health states. On the horizontal axis, the average value of the crosswalk and EQ-VT value set is shown for each health state

Figure 2 shows a Bland–Altman plot for the existing EQ-5D-5L crosswalk value set and the EQ-VT value set. The EQ-VT produces lower values on average (mean EQ-VT 0.386, while this is 0.524 in the crosswalk). The EQ-VT value set had a higher number of negative values (275 states or 8.8% versus 21 states or 0.7%). The mean absolute difference between the value set and the crosswalk set was 0.157 and the correlation coefficient between the two sets was 0.879. The EQ-VT value set had a wider range (-0.563 to 1.000) than the crosswalk set (-0.163 to 1.000).

## Discussion

### Main findings

This study produced a set of EQ-5D-5L values that directly represent the preferences of the Trinidad and Tobago adult population and that can now be used in clinical and economic applications in Trinidad and Tobago as well as in other Caribbean countries for applications in which the Trinidad and Tobago crosswalk values might have been used. Furthermore, the current study is the first one to use a mixed logit model to analyse the DCE data, owing to the use of a larger health state design for the DCE task. The mixed logit models produced similar results to the cTTO-only and hybrid models. The existing crosswalk value set was shown to be considerably different from the EQ-VT value set.

### Interpretation

There are two main drivers of differences between values in EQ-5D value sets: differences in scale and differences in the magnitudes of the coefficients of the underlying utility function relative to each other. Differences in scale between EQ-5D-3L value sets and EQ-VT based value sets have been observed in other studies [26]. For differences in scale, the lower values and the increase in the number of negative values in the new value set can be explained by several factors. There could have been greater willingness to trade life-years in the EQ-VT protocol than in the modified MVH Time Trade Off (TTO) protocol that was used to recalibrate the DCE data to a 0 (dead) to 1 (full health) scale in the 2016 EQ-5D-3L valuation study [5]. In the 2016 study, 10% of the respondents who completed all of the TTO tasks were non-traders (assigning the full health value to all states). In this (2023) study this fell to 0.2% (only 2 non-traders). This would in part be associated with the quality control protocol in EQ-VT which ensures that interviewers explain the cTTO tasks to each respondent, thereby promoting a better understanding of the cTTO tasks on behalf of the respondents. Generally, the introduction of the EQ-VT data quality control protocol ensures that respondents were explained all elements of the cTTO task, leading to more reliable data compared to TTO studies that do not employ a quality control strategy [24]. Further, the use of a pilot phase during the data collection may have improved the quality of the collected data which may impact the outcomes of the study as well [26]. Lastly, the method used to value health states with negative values has changed compared to the Measurement and Valuation of Health (MVH) protocol that was followed in the 2016 valuation study, which may affect the values elicited for those states.

The differences in patterns among the coefficients of the crosswalk and EQ-VT value sets could be associated with social change over the 7 year period: e.g. greater awareness of mental health may have influenced the anxiety/depression coefficients and the lock down associated with covid over 2020–2022 may have brought increased salience of usual activities to the respondents [27]. Such changes may highlight the need for revisiting/updating EQ-5D value sets. Further, the crosswalk algorithm was developed based on responses from European respondents. It is possible that T&T respondents respond differently to EQ-5D-3L and EQ-5D-5L, which may exacerbate any differences in value sets. Lastly, the descriptive systems are different between the EQ-5D-3L and the EQ-5D-5L in the mobility dimension, with level 3 mobility being defined as “confined to bed” in the EQ-5D-3L, while level 5 for mobility in the EQ-5D-5L is defined as “unable to walk about”. Directly valuing “confined to bed” may lead to a higher willingness to trade life years as compared to valuing “unable to walk”, as it may be perceived as being more severe [28]. This may (partially) explain differences observed between the crosswalk and directly evaluated EQ-5D-5L value sets, as for the crosswalk, the value for health states with level 4 or 5 problems on mobility are inferred from the value assigned to “being confined to bed”.

Compared to the USA which also used EQ-VT, the Trinidad and Tobago level 5 coefficients follow a similar pattern with pain/discomfort having the largest coefficients in both values sets (0.480 versus 0.414 for the USA) and usual activities having the smallest coefficients (0.219 and 0.255 respectively) [29]. At level 5, both value sets show the same ranking of coefficients (smallest to largest): UA, SC, AD, MO, PD. However at other levels the rankings are not the same, for example for the level 1 coefficients the Trinidad and Tobago value set has: UA, AD, SC, MO, PD whereas for the USA this is AD, PD, UA, SC, MO. State 55555 has values of -0.573 (USA) and -0.563 (Trinidad and Tobago). Such differences in ranking and scale show the importance of using local health-state values to inform resource allocation decisions.

This was the first use of mixed logit models in EQ-VT (due to the use of a new DCE design, which has more choice tasks per respondent, which allows for the mixed logit model to be identified). The current study shows that an expanded health state design for the DCE task allows us to estimate mixed logit models on EQ-VT data, which are theoretically superior to the standard conditional logit model. The results of the mixed logit models were, after rescaling, similar to those of the cTTO-only and hybrid models.

### Limitations and strengths

This study had some minor limitations in the sampling. Females in the 35–44 and 55–64 age groups were slightly over-represented. More educated groups were also over-represented but this has been found to have little or no impact on EQ-5D valuation results [30, 31]. Another limitation is that there were some protocol compliance issues with some of the interviewers. Some of these could be solved during the pilot phase but some issues persisted beyond, which had to be resolved during the data collection. Furthermore there were also some interviewer effects suggesting that there may have been some differences in how interviewers conducted their interviews, although this could also be associated with demographic differences in the respondents interviewed by each interviewer.

There are several strengths and contributions of this study. First, our study design allowed the use of mixed logit models to analyse the DCE data; the first country to do so for the EQ-5D-5L using the EQ-VT protocol. Second, the study created an updated set of values for the Trinidad and Tobago population that will replace the crosswalk values. Lastly, the crosswalk was originally created to provide interim value sets for countries that had EQ-5D-3L value sets. Crosswalk sets can also be used in resource-constrained settings to allow users time to build capacity in working with health outcomes. This would facilitate the use of EQ-5D without the resource commitment for EQ-VT, until an EQ-5D-5L valuation study can be undertaken. This study gives users in Trinidad and Tobago the opportunity to move to an updated value set that represents the preferences of the population as of 2022.

## Conclusion

This study has produced a state of the art EQ-5D-5L value set for Trinidad and Tobago that can now be used in clinical and resource-allocation decision-making. Changes in the value sets for Trinidad and Tobago over the period 2016 to 2022 highlight the need for revising EQ-5D value sets to ensure they are developed using the highest standard of current practice, and represent the current preferences of the national population. Furthermore, this value set represents the first value set developed using the EQ-VT protocol in the Caribbean and may be used as a reference case for countries in the region with similar population characteristics.

## Data Availability

The data that support the findings of this study are available from the corresponding author, but restrictions apply to the availability of these data, which are not publicly available. The data are, however, available from the authors upon reasonable request.

## Appendix A

### Full modelling results

**Table 5:** Results of modeling cTTO responses. NC indicates that no constant was estimated in the model

	**Randomintercept**			**Random intercept Tobit**			**Heteroskedastic**			**Heteroskedastic (NC)**			**Heteroskedastic tobit (NC)**		
coefficients	Beta	SE	*P*-value	Beta	SE	*P*-value	Beta	SE	*P*-value	Beta	SE	*P*-value	Beta	SE	*P*-value
**mo2**	0.018	0.012	0.118	0.011	0.012	0.398	0.018	0.008	0.016	0.018	0.006	0.004	0.014	0.007	0.048
**mo3**	0.049	0.012	0.000	0.035	0.013	0.006	0.063	0.013	0.000	0.063	0.013	0.000	0.058	0.013	0.000
**mo4**	0.127	0.013	0.000	0.112	0.014	0.000	0.164	0.014	0.000	0.164	0.013	0.000	0.162	0.014	0.000
**mo5**	0.323	0.012	0.000	0.334	0.013	0.000	0.344	0.013	0.000	0.344	0.013	0.000	0.357	0.014	0.000
**sc2**	0.028	0.012	0.018	0.024	0.012	0.050	0.031	0.007	0.000	0.031	0.006	0.000	0.029	0.007	0.000
**sc3**	0.055	0.013	0.000	0.051	0.014	0.000	0.076	0.011	0.000	0.076	0.011	0.000	0.074	0.011	0.000
**sc4**	0.160	0.013	0.000	0.156	0.014	0.000	0.157	0.013	0.000	0.157	0.013	0.000	0.159	0.013	0.000
**sc5**	0.257	0.012	0.000	0.283	0.013	0.000	0.209	0.011	0.000	0.208	0.011	0.000	0.221	0.012	0.000
**ua2**	0.036	0.012	0.003	0.033	0.013	0.009	0.018	0.007	0.012	0.018	0.006	0.001	0.016	0.007	0.021
**ua3**	0.083	0.013	0.000	0.083	0.014	0.000	0.086	0.011	0.000	0.086	0.011	0.000	0.087	0.011	0.000
**ua4**	0.159	0.013	0.000	0.157	0.014	0.000	0.144	0.012	0.000	0.144	0.011	0.000	0.145	0.011	0.000
**ua5**	0.235	0.012	0.000	0.260	0.013	0.000	0.202	0.012	0.000	0.202	0.012	0.000	0.216	0.013	0.000
**pd2**	0.036	0.011	0.001	0.030	0.011	0.008	0.028	0.006	0.000	0.029	0.005	0.000	0.026	0.006	0.000
**pd3**	0.110	0.013	0.000	0.108	0.014	0.000	0.104	0.012	0.000	0.104	0.012	0.000	0.102	0.013	0.000
**pd4**	0.316	0.012	0.000	0.323	0.012	0.000	0.311	0.012	0.000	0.311	0.012	0.000	0.316	0.013	0.000
**pd5**	0.517	0.013	0.000	0.535	0.013	0.000	0.511	0.014	0.000	0.511	0.014	0.000	0.541	0.016	0.000
**ad2**	0.023	0.013	0.068	0.020	0.013	0.143	0.026	0.006	0.000	0.026	0.005	0.000	0.024	0.006	0.000
**ad3**	0.065	0.014	0.000	0.055	0.015	0.000	0.067	0.012	0.000	0.067	0.012	0.000	0.057	0.012	0.000
**ad4**	0.178	0.013	0.000	0.175	0.014	0.000	0.172	0.012	0.000	0.172	0.012	0.000	0.168	0.012	0.000
**ad5**	0.272	0.012	0.000	0.283	0.013	0.000	0.264	0.011	0.000	0.264	0.011	0.000	0.272	0.011	0.000
**Constant**	-0.012	0.015	0.411	-0.010	0.015	0.527	0.001	0.007	0.921	N/A	N/A	N/A	N/A	N/A	N/A
**AIC**	9609.975			11,562.85			8269.441			8267.45			9265.768		
**BIC**	9777.562			11,730.43			8575.468			8566.192			9571.795		
**MAE (total)**	0.292			0.292			0.294			0.294			0.295		
**MAE(86)**	0.040			0.049			0.038			0.038			0.043		
**v(55555)**	-0.593			-0.686			-0.530			-0.530			-0.607

**Table 6:** DCE logit modelling results. Rescaled using the scale length for the hybrid heteroskedastic model. Rescaling factor: 0.0641

	**Conditional logit**			**Mixed logit**			
	Beta	SE	*P*-value	Mean Beta	SE	*P*-value	Mean Beta (rescaled)
**mo2**	0.341	0.053	0.000	0.746	0.098	0.000	0.048
**mo3**	0.706	0.053	0.000	1.51	0.123	0.000	0.097
**mo4**	1.380	0.069	0.000	3.165	0.179	0.000	0.203
**mo5**	2.524	0.091	0.000	5.701	0.298	0.000	0.365
**sc2**	0.152	0.052	0.004	0.473	0.110	0.000	0.030
**sc3**	0.488	0.054	0.000	1.236	0.111	0.000	0.079
**sc4**	1.006	0.063	0.000	2.427	0.150	0.000	0.156
**sc5**	1.584	0.073	0.000	3.773	0.197	0.000	0.242
**ua2**	0.088	0.052	0.090	0.173	0.108	0.109	0.011
**ua3**	0.430	0.052	0.000	0.961	0.117	0.000	0.062
**ua4**	0.981	0.060	0.000	2.152	0.140	0.000	0.138
**ua5**	1.450	0.068	0.000	3.186	0.176	0.000	0.204
**pd2**	0.555	0.053	0.000	1.259	0.111	0.000	0.081
**pd3**	1.020	0.057	0.000	2.372	0.139	0.000	0.152
**pd4**	2.178	0.083	0.000	5.031	0.238	0.000	0.322
**pd5**	3.218	0.104	0.000	7.730	0.340	0.000	0.495
**ad2**	0.102	0.052	0.049	0.263	0.119	0.027	0.017
**ad3**	0.515	0.056	0.000	1.237	0.137	0.000	0.079
**ad4**	1.064	0.073	0.000	2.436	0.192	0.000	0.156
**ad5**	1.735	0.087	0.000	3.988	0.246	0.000	0.256
**AIC**	16424.36			15471.58			
**BIC**	16573.73			17348.8			
**MAE (total)**	N/A			N/A			0.303
**MAE(86 states)**	N/A			N/A			0.053
**v(55555)**	10.51			24.38			-0.563

**Table 7:** Hybrid modelling results

	**Hybrid**			**Hybrid tobit**			**Hybrid heteroskedastic**			**Hybrid heteroskedastic Tobit**		
coefficient	Beta	SE	*P*-value	Beta	SE	*P*-value	Beta	SE	P-value	Beta	SE	*P*-value
**mo2**	0.034	0.007	0.000	0.029	0.007	0.000	0.029	0.005	0.000	0.027	0.005	0.000
**mo3**	0.086	0.007	0.000	0.084	0.007	0.000	0.084	0.006	0.000	0.085	0.006	0.000
**mo4**	0.184	0.007	0.000	0.185	0.007	0.000	0.184	0.006	0.000	0.187	0.006	0.000
**mo5**	0.363	0.007	0.000	0.379	0.008	0.000	0.357	0.007	0.000	0.368	0.007	0.000
**sc2**	0.018	0.006	0.004	0.014	0.007	0.041	0.024	0.005	0.000	0.024	0.004	0.000
**sc3**	0.065	0.007	0.000	0.063	0.007	0.000	0.071	0.006	0.000	0.072	0.006	0.000
**sc4**	0.146	0.007	0.000	0.146	0.007	0.000	0.147	0.006	0.000	0.150	0.006	0.000
**sc5**	0.233	0.007	0.000	0.245	0.007	0.000	0.225	0.006	0.000	0.232	0.006	0.000
**ua2**	0.006	0.006	0.388	0.001	0.007	0.926	0.012	0.004	0.007	0.011	0.004	0.007
**ua3**	0.061	0.006	0.000	0.060	0.007	0.000	0.065	0.006	0.000	0.065	0.006	0.000
**ua4**	0.141	0.007	0.000	0.143	0.007	0.000	0.142	0.006	0.000	0.146	0.006	0.000
**ua5**	0.219	0.007	0.000	0.231	0.007	0.000	0.212	0.006	0.000	0.219	0.006	0.000
**pd2**	0.066	0.006	0.000	0.065	0.007	0.000	0.046	0.004	0.000	0.044	0.004	0.000
**pd3**	0.139	0.007	0.000	0.142	0.007	0.000	0.127	0.006	0.000	0.128	0.006	0.000
**pd4**	0.318	0.007	0.000	0.329	0.007	0.000	0.304	0.006	0.000	0.311	0.007	0.000
**pd5**	0.486	0.008	0.000	0.511	0.008	0.000	0.464	0.007	0.000	0.480	0.008	0.000
**ad2**	0.011	0.007	0.084	0.007	0.007	0.342	0.020	0.004	0.000	0.020	0.004	0.000
**ad3**	0.069	0.007	0.000	0.065	0.007	0.000	0.075	0.006	0.000	0.074	0.006	0.000
**ad4**	0.156	0.007	0.000	0.156	0.007	0.000	0.160	0.006	0.000	0.161	0.006	0.000
**ad5**	0.259	0.007	0.000	0.268	0.007	0.000	0.257	0.006	0.000	0.264	0.006	0.000
**AIC**	28194.11			30170.3			24743.61			25755.71		
**BIC**	28371.76			30347.94			25082.75			26094.86		
**MAE (total)**	0.297			0.297			0.296			0.297		
**MAE(86 states)**	0.045			0.052			0.042			0.044		
**v(55555)**	-0.560			-0.634			-0.515			-0.563

**Table 8:** leave-out analyses. MAE per model

MAE	Leave out state	Leave out block
**Random intercept**	0.054	0.051
**Random intercept Tobit**	0.065	0.060
**Heteroskedastic**	0.050	0.052
**Heteroskedastic (no constant)**	0.049	0.052
**Heteroskedastic Tobit**	0.056	0.042
**Hybrid**	0.049	0.051
**Hybrid Tobit**	0.057	0.054
**Hybrid heteroskedastic**	0.046	0.051
**Hybrid heteroskedastic Tobit**	0.049	0.050

## Appendix B

### Technical details on modelling

This technical appendix provides an overview of the functional forms of the estimated models. All models were estimated as 20-parameter models, which means that the observed cTTO responses are modelled as a function of the level-dimension combinations of the EQ-5D-5L health state being valued. As there are 5 dimensions, with 5 levels each, each health state is represented by 20 dummy coded variables, indicating whether a particular level of problems is present on a dimension, with level 1 being the reference category for each dimension. For example, the dummy variable $MO4$ then represents having level 4 problems with mobility, or in other words, severe problems with walking about. The associated coefficient ${\beta }_{3}$ represents the decrement in value for having this level of problems on mobility.

### Random intercept model

$${U}_{ij}={\beta }_{0}+{\beta }_{1}{MO2}_{j}+{\beta }_{2}{MO3}_{j}+{\beta }_{3}{MO4}_{j}+{\beta }_{4}{MO5}_{j}+{\beta }_{5}{SC2}_{j}+{\beta }_{6}{SC3}_{j}+{\beta }_{7}{SC4}_{j}+{\beta }_{8}{SC5}_{j}+{\beta }_{9}{UA2}_{j}+{\beta }_{10}{UA3}_{j}+{\beta }_{11}{UA4}_{j}+{\beta }_{12}{UA5}_{j}+{\beta }_{13}{PD2}_{j}+{\beta }_{14}{PD3}_{j}+{\beta }_{15}{PD4}_{j}+{\beta }_{16}{PD5}_{j}+{\beta }_{17}{AD2}_{j}+{\beta }_{18}{AD3}_{j}+{\beta }_{19}{AD4}_{j}+{\beta }_{20}{AD5}_{j}+{\varepsilon }_{ij}+{\mu }_{i}$$

The random intercept model models the utility ${U}_{ij}$ assigned by respondents $i$ to health state $j.$ Here, ${\beta }_{0}$ represents the fixed intercept, while ${\mu }_{i}$ represents a respondent-level intercept parameter, assumed to be normally distributed with mean 0, ${\mu }_{j} \sim N(0,{\sigma }_{\mu }^{2})$. ${\varepsilon }_{ij}$ is the error term for health state $j$ valued by respondent $i$, and is assumed to be distributed as ${\varepsilon }_{ij} \sim N(0,{\sigma }_{j}^{2})$.

### Random intercept Tobit model

The random intercept Tobit regression takes the same functional form as that of the random intercept model. However, the data is also assumed to be “left censored” at -1, which means that no values beyond -1 are observed due to the fact that this is not possible in the cTTO task, but that it may be the case that some respondents would have assigned lower values to some health states in case this would have been possible in the cTTO task. Therefore, the following link function is assumed:

$$U=\left\{\begin{array}{c}U" if U">-1\\ -1 if U"<-1\end{array}\right.$$

Here, $U$ is the observed cTTO response, while $U"$ is a latent variable that is assumed to be underlying of $U$, and the tobit model adjusts the parameter estimates based on the probability of the latent variable $U"$ being smaller than the threshold -1 [32, 33].

### Heteroskedastic model and heteroskedastic Tobit model

Two models were estimated to correct for the heteroskedastic nature of the data; the fact that the error term is often not normally distributed in 20-parameter models for cTTO data. Instead, the error terms is usually larger for more severe health states, and smaller for those health states which are relatively mild. Therefore, the error term ${\varepsilon }_{j}$ of the equation below, which was modelled as a function of the 20 dummy variables of the model itself, ${MO2}_{j}$ to ${AD5}_{j}$.

$${U}_{j}={\beta }_{0}+{\beta }_{1}{MO2}_{j}+{\beta }_{2}{MO3}_{j}+{\beta }_{3}{MO4}_{j}+{\beta }_{4}{MO5}_{j}+{\beta }_{5}{SC2}_{j}+{\beta }_{6}{SC3}_{j}+{\beta }_{7}{SC4}_{j}+{\beta }_{8}{SC5}_{j}+{\beta }_{9}{UA2}_{j}+{\beta }_{10}{UA3}_{j}+{\beta }_{11}{UA4}_{j}+{\beta }_{12}{UA5}_{j}+{\beta }_{13}{PD2}_{j}+{\beta }_{14}{PD3}_{j}+{\beta }_{15}{PD4}_{j}+{\beta }_{16}{PD5}_{j}+{\beta }_{17}{AD2}_{j}+{\beta }_{18}{AD3}_{j}+{\beta }_{19}{AD4}_{j}+{\beta }_{20}{AD5}_{j}+{\varepsilon }_{j}$$

The variance function for the error term is then represented as following:

$${\sigma }_{j}^{2}=\text{exp}({\gamma }_{0}+{\gamma }_{1}{MO2}_{j}+{\gamma }_{2}{MO3}_{j}+{\gamma }_{3}{MO4}_{j}+{\gamma }_{4}{MO5}_{j}+{\gamma }_{5}{SC2}_{j}+{\gamma }_{6}{SC3}_{j}+{\gamma }_{7}{SC4}_{j}+{\gamma }_{8}{SC5}_{j}+{\gamma }_{9}{UA2}_{j}+{\gamma }_{10}{UA3}_{j}+{\gamma }_{11}{UA4}_{j}+{\gamma }_{12}{UA5}_{j}+{\gamma }_{13}{PD2}_{j}+{\gamma }_{14}{PD3}_{j}+{\gamma }_{15}{PD4}_{j}+{\gamma }_{16}{PD5}_{j}+{\gamma }_{17}{AD2}_{j}+{\gamma }_{18}{AD3}_{j}+{\gamma }_{19}{AD4}_{j}+{\gamma }_{20}{AD5}_{j})$$

For the Tobit model corrected for heteroskedasticity, the same functional form for both the model as well as the variance of the error term is assumed, with the additional Tobit link function as assumed in the random intercept Tobit model.

### Conditional logit model

The conditional logit model assumes that an individual has a utility function $U\left(X\right),$ which is based on a fixed part, as well as a stochastic part [34]. The fixed part in our case takes the form:

$${U}_{j}={\beta }_{1}{MO2}_{j}+{\beta }_{2}{MO3}_{j}+{\beta }_{3}{MO4}_{j}+{\beta }_{4}{MO5}_{j}+{\beta }_{5}{SC2}_{j}+{\beta }_{6}{SC3}_{j}+{\beta }_{7}{SC4}_{j}+{\beta }_{8}{SC5}_{j}+{\beta }_{9}{UA2}_{j}+{\beta }_{10}{UA3}_{j}+{\beta }_{11}{UA4}_{j}+{\beta }_{12}{UA5}_{j}+{\beta }_{13}{PD2}_{j}+{\beta }_{14}{PD3}_{j}+{\beta }_{15}{PD4}_{j}+{\beta }_{16}{PD5}_{j}+{\beta }_{17}{AD2}_{j}+{\beta }_{18}{AD3}_{j}+{\beta }_{19}{AD4}_{j}+{\beta }_{20}{AD5}_{j}$$

If a respondent chooses between health states $j$ and $k$, then probability of choosing health state $j$ is modelled as:

$$P\left(j|X,k\right)=\frac{{e}^{{U}_{j}(\beta {X}_{j})}}{{e}^{{U}_{j}(\beta {X}_{j})}+{e}^{{U}_{k}(\beta {X}_{k})}}$$

Here, $\beta X$ represents the weights $\beta$ associated to the vector of attributes of the health states $X$ as outlined in the fixed part of the utility function. The choice probabilities are subsequently modelled over the whole population, ignoring that each respondent provides multiple datapoints.

### Mixed logit model

In the mixed logit model, in contrast to the conditional logit, the $\beta$ parameters are allowed to vary between respondents. This means that the fixed portion of the utility function takes the form:

$${U}_{ij}={\beta }_{i1}{MO2}_{ij}+{\beta }_{i2}{MO3}_{ij}+{\beta }_{i3}{MO4}_{ij}+{\beta }_{i4}{MO5}_{ij}+{\beta }_{i5}{SC2}_{ij}+{\beta }_{i6}{SC3}_{ij}+{\beta }_{i7}{SC4}_{ij}+{\beta }_{i8}{SC5}_{ij}+{\beta }_{i9}{UA2}_{ij}+{\beta }_{i10}{UA3}_{ij}+{\beta }_{i11}{UA4}_{ij}+{\beta }_{i12}{UA5}_{ij}+{\beta }_{i13}{PD2}_{ij}+{\beta }_{i14}{PD3}_{ij}+{\beta }_{i15}{PD4}_{ij}+{\beta }_{i16}{PD5}_{ij}+{\beta }_{i17}{AD2}_{ij}+{\beta }_{i18}{AD3}_{ij}+{\beta }_{i19}{AD4}_{ij}+{\beta }_{i20}{AD5}_{ij}$$

This means that respondent $i$’s choice probability is modelled as follows:

$${P}_{i}\left(j|X,k\right)=\int \frac{{e}^{{U}_{ij}\left({\beta }_{i}{X}_{ij}\right)}}{{e}^{{U}_{ij}\left({\beta }_{i}{X}_{ij}\right)}+{e}^{{U}_{ik}\left({\beta }_{i}{X}_{ik}\right)}}f\left({\beta }_{i}|\theta \right)d({\beta }_{i})$$

Here, $f\left({\beta }_{i}|\theta \right)$ represents the density function of ${\beta }_{i}.$

### Hybrid models

For the hybrid models, the following functional form is again assumed for the cTTO data:

$${U}_{j}={\beta }_{0}+{\beta }_{1}{MO2}_{j}+{\beta }_{2}{MO3}_{j}+{\beta }_{3}{MO4}_{j}+{\beta }_{4}{MO5}_{j}+{\beta }_{5}{SC2}_{j}+{\beta }_{6}{SC3}_{j}+{\beta }_{7}{SC4}_{j}+{\beta }_{8}{SC5}_{j}+{\beta }_{9}{UA2}_{j}+{\beta }_{10}{UA3}_{j}+{\beta }_{11}{UA4}_{j}+{\beta }_{12}{UA5}_{j}+{\beta }_{13}{PD2}_{j}+{\beta }_{14}{PD3}_{j}+{\beta }_{15}{PD4}_{j}+{\beta }_{16}{PD5}_{j}+{\beta }_{17}{AD2}_{j}+{\beta }_{18}{AD3}_{j}+{\beta }_{19}{AD4}_{j}+{\beta }_{20}{AD5}_{j}+{\varepsilon }_{j}$$

These models can be estimated using the Tobit link function to account for left-censoring, as well as modelling the variance of the error term, as specified for the heteroskedastic model. For the DCE data, a conditional logit model type of functional form is assumed. The data is then modelled jointly. For more details on the likelihood function of the hybrid model, see Ramos-Goni et al. [35].

## Appendix C

### DCE health state design

**Table:**

			**A**					**B**			
Block	**Choice situation**	**mo**	**sc**	**ua**	**pd**	**ad**	**mo**	**sc**	**ua**	**pd**	**ad**
**1**	1	3	1	1	1	4	1	1	1	2	3
**1**	2	4	1	5	3	1	5	2	4	3	1
**1**	3	2	3	3	4	2	4	2	2	4	2
**1**	4	2	1	2	3	3	2	4	2	2	2
**1**	5	2	5	4	5	5	5	5	3	4	5
**1**	6	3	4	5	5	3	1	1	5	5	5
**1**	7	3	5	4	1	2	1	4	4	1	4
**1**	8	4	4	3	1	5	4	5	3	2	4
**1**	9	4	1	4	4	4	2	2	5	4	4
**1**	10	5	1	2	5	1	4	2	3	5	1
**1**	11	3	3	1	2	5	1	3	1	5	2
**1**	12	2	3	2	4	3	5	3	5	1	3
**2**	13	1	5	2	3	1	1	1	3	4	1
**2**	14	5	4	1	5	1	5	4	2	4	2
**2**	15	3	2	3	2	2	3	2	1	4	1
**2**	16	3	3	4	3	4	4	3	5	3	3
**2**	17	2	5	1	3	2	2	5	2	2	1
**2**	18	2	4	5	1	4	3	2	5	1	5
**2**	19	5	2	1	2	3	2	2	5	2	5
**2**	20	3	3	1	3	5	1	2	4	3	5
**2**	21	5	3	4	2	1	3	4	4	2	3
**2**	22	4	4	3	3	5	4	4	1	5	2
**2**	23	4	5	2	1	4	5	4	2	1	5
**2**	24	1	2	2	5	4	1	5	4	5	1
**3**	25	3	5	5	4	4	4	5	4	4	3
**3**	26	5	1	4	2	2	5	1	3	4	3
**3**	27	1	3	3	2	1	1	2	3	1	2
**3**	28	5	2	1	3	4	5	5	3	3	2
**3**	29	1	5	5	3	1	1	3	5	4	2
**3**	30	2	4	1	2	4	2	3	4	3	4
**3**	31	3	4	3	5	3	3	5	1	4	3
**3**	32	2	1	2	1	4	3	1	1	1	5
**3**	33	2	3	5	5	5	3	3	2	5	4
**3**	34	4	2	3	2	1	4	4	1	1	1
**3**	35	2	1	2	2	5	4	1	5	2	1
**3**	36	4	3	2	1	5	5	3	4	1	3
**4**	37	5	2	5	3	2	3	1	5	5	2
**4**	38	1	2	1	4	2	1	3	1	3	4
**4**	39	3	4	2	3	1	3	3	3	3	2
**4**	40	1	5	2	5	3	2	4	4	5	3
**4**	41	2	2	3	1	3	2	1	5	1	2
**4**	42	4	2	4	4	5	5	4	3	4	5
**4**	43	5	5	4	4	4	5	2	5	5	4
**4**	44	3	1	3	3	5	4	1	3	2	4
**4**	45	2	5	2	5	3	3	5	5	2	3
**4**	46	4	4	4	5	2	2	1	4	5	4
**4**	47	1	3	2	4	5	1	5	1	1	5
**4**	48	1	4	1	2	5	1	4	5	1	3
**5**	49	1	1	3	3	2	2	1	2	3	3
**5**	50	5	3	1	5	1	4	4	1	4	1
**5**	51	2	4	4	3	1	2	3	5	4	1
**5**	52	5	5	3	1	5	3	5	3	5	4
**5**	53	1	2	4	4	3	4	2	5	2	3
**5**	54	3	2	1	1	3	2	5	1	1	1
**5**	55	3	3	2	2	5	5	2	2	2	4
**5**	56	4	3	4	2	2	3	5	1	2	2
**5**	57	5	4	3	5	3	2	2	3	5	5
**5**	58	1	4	4	2	2	1	4	3	3	1
**5**	59	4	5	2	3	4	2	4	5	3	4
**5**	60	5	1	3	2	4	5	1	5	3	2
**6**	61	3	3	2	1	4	5	2	2	1	3
**6**	62	4	1	5	4	5	4	5	2	4	4
**6**	63	1	3	1	5	3	4	5	1	3	3
**6**	64	5	3	4	3	1	2	1	4	4	1
**6**	65	1	5	2	2	5	1	2	4	2	4
**6**	66	2	3	5	3	1	3	4	5	2	1
**6**	67	2	1	1	2	2	3	1	1	4	1
**6**	68	4	4	3	3	2	4	5	4	1	2
**6**	69	5	3	2	4	2	4	3	2	5	3
**6**	70	4	2	1	5	5	4	1	5	5	3
**6**	71	4	1	2	1	1	1	5	3	1	1
**6**	72	3	2	5	4	5	1	4	4	4	5
**7**	73	3	2	4	1	5	3	5	4	3	3
**7**	74	4	1	4	5	4	5	4	3	5	4
**7**	75	4	4	1	3	2	2	5	1	4	2
**7**	76	2	3	3	1	3	1	5	3	1	2
**7**	77	3	2	2	3	1	4	2	3	1	1
**7**	78	5	4	4	4	5	5	1	5	5	5
**7**	79	5	3	1	1	4	2	4	1	2	4
**7**	80	1	2	4	5	2	4	2	2	4	2
**7**	81	1	2	5	2	3	2	3	5	2	2
**7**	82	3	3	3	2	3	2	1	3	3	3
**7**	83	3	1	3	4	4	5	1	1	2	4
**7**	84	5	5	2	5	1	5	5	5	3	4
**8**	85	3	5	4	1	5	4	3	4	3	5
**8**	86	1	2	5	4	3	1	4	3	4	4
**8**	87	2	1	4	4	1	1	1	2	5	1
**8**	88	3	3	5	5	2	5	5	5	2	2
**8**	89	1	2	3	3	5	2	2	2	3	4
**8**	90	3	1	4	3	3	1	1	1	4	3
**8**	91	4	5	5	1	2	5	4	5	1	1
**8**	92	5	3	2	1	2	4	4	2	1	1
**8**	93	2	4	2	2	5	1	4	5	2	4
**8**	94	3	2	2	4	1	2	2	1	5	1
**8**	95	5	2	1	3	2	5	3	1	2	3
**8**	96	3	4	5	5	2	3	2	3	5	4
**9**	97	5	1	1	3	5	4	3	1	2	5
**9**	98	4	5	4	3	1	3	5	4	2	3
**9**	99	2	4	2	5	5	2	5	4	5	2
**9**	100	5	3	1	4	5	2	5	2	4	5
**9**	101	3	1	1	1	5	5	1	2	1	3
**9**	102	4	4	3	4	1	2	3	3	4	4
**9**	103	1	2	2	1	5	1	4	3	1	4
**9**	104	4	3	5	4	3	4	2	5	2	4
**9**	105	1	3	4	5	1	1	3	5	4	2
**9**	106	3	1	3	5	2	3	1	4	4	4
**9**	107	2	2	3	1	3	2	4	2	1	2
**9**	108	5	5	5	3	3	5	5	3	4	1
**10**	109	1	5	4	1	3	1	1	4	2	5
**10**	110	3	4	4	2	3	3	4	3	4	2
**10**	111	1	5	1	4	4	3	5	1	5	2
**10**	112	3	4	2	3	1	3	2	5	1	1
**10**	113	5	3	3	1	1	3	3	5	4	1
**10**	114	4	1	3	2	3	5	1	2	2	1
**10**	115	4	3	4	5	1	4	2	2	5	3
**10**	116	3	1	3	1	1	4	1	1	1	2
**10**	117	1	5	5	3	5	1	4	5	5	4
**10**	118	2	1	4	4	5	2	3	5	3	5
**10**	119	4	3	2	3	4	2	4	1	3	4
**10**	120	5	2	4	3	5	5	5	1	2	5
**11**	121	2	2	3	1	3	2	2	5	3	2
**11**	122	3	3	3	2	2	1	3	2	2	4
**11**	123	1	1	2	5	2	1	1	5	1	4
**11**	124	2	4	4	1	1	2	5	1	2	1
**11**	125	5	2	1	4	1	3	2	1	5	4
**11**	126	4	2	4	2	2	2	5	4	1	2
**11**	127	5	4	5	1	3	5	4	3	3	4
**11**	128	2	4	4	2	3	3	4	2	4	3
**11**	129	1	4	1	2	1	1	3	1	3	3
**11**	130	5	2	3	5	4	4	1	3	5	5
**11**	131	5	1	4	1	2	2	2	4	4	2
**11**	132	4	4	1	3	3	4	3	1	1	5
**12**	133	2	3	4	5	1	3	3	3	5	2
**12**	134	4	4	2	4	3	4	3	5	4	4
**12**	135	3	5	2	2	4	1	5	3	2	5
**12**	136	2	3	3	5	5	1	2	5	5	5
**12**	137	5	5	2	5	2	3	5	2	4	5
**12**	138	5	1	5	4	1	4	5	5	2	1
**12**	139	1	1	1	4	3	1	1	2	3	5
**12**	140	2	4	5	5	5	5	5	1	5	5
**12**	141	3	2	4	5	4	4	2	2	4	4
**12**	142	2	5	5	1	4	4	3	4	1	4
**12**	143	1	3	5	3	3	1	2	3	3	4
**12**	144	4	1	2	5	2	5	4	2	2	2
**13**	145	2	5	3	3	5	4	5	4	3	4
**13**	146	3	1	5	3	3	4	1	2	3	5
**13**	147	2	4	1	3	2	1	3	1	4	2
**13**	148	5	1	4	3	3	5	2	4	2	2
**13**	149	3	2	2	5	1	3	4	1	4	1
**13**	150	5	3	3	1	4	3	3	4	1	5
**13**	151	2	3	3	2	3	2	2	1	4	3
**13**	152	5	1	4	2	1	4	5	5	2	1
**13**	153	3	5	3	1	1	2	3	5	1	1
**13**	154	3	4	2	5	3	2	5	4	5	3
**13**	155	1	5	2	3	2	4	2	3	3	2
**13**	156	1	1	2	2	3	1	3	2	3	2
**14**	157	4	2	1	1	1	1	4	1	1	3
**14**	158	2	1	4	1	2	4	1	2	1	3
**14**	159	5	3	2	4	4	5	2	4	4	3
**14**	160	4	1	1	5	4	4	3	1	4	5
**14**	161	1	1	3	4	4	1	3	3	5	1
**14**	162	5	2	2	2	5	4	5	3	2	5
**14**	163	3	4	1	3	5	2	4	3	2	5
**14**	164	5	3	5	4	3	5	4	5	1	5
**14**	165	3	5	2	2	2	3	3	1	2	4
**14**	166	1	5	5	5	5	4	4	5	5	2
**14**	167	2	2	5	3	1	3	2	4	2	1
**14**	168	1	5	5	2	4	5	5	3	1	4
**15**	169	3	3	3	3	3	3	1	2	3	5
**15**	170	2	5	2	4	3	3	5	5	4	1
**15**	171	1	4	3	2	1	1	3	1	3	1
**15**	172	5	4	4	5	2	5	4	5	2	3
**15**	173	1	3	4	4	4	4	2	4	3	4
**15**	174	2	5	3	4	4	2	4	5	4	2
**15**	175	4	5	3	4	3	4	3	3	2	5
**15**	176	2	4	1	1	4	2	5	1	2	2
**15**	177	1	1	3	1	5	1	1	4	2	4
**15**	178	3	1	1	5	3	4	1	1	4	1
**15**	179	4	2	1	5	5	5	2	4	3	5
**15**	180	5	2	2	1	2	2	2	4	1	5
**16**	181	3	4	3	3	4	3	4	2	4	3
**16**	182	5	3	1	5	3	4	2	4	5	3
**16**	183	3	1	3	2	2	1	1	5	3	2
**16**	184	2	5	3	5	1	3	3	4	5	1
**16**	185	2	1	4	5	4	4	1	5	2	4
**16**	186	5	2	2	2	1	4	4	2	1	1
**16**	187	1	4	2	5	2	1	4	4	4	3
**16**	188	5	5	4	2	5	5	2	3	4	5
**16**	189	2	4	1	2	4	3	5	1	1	4
**16**	190	5	1	5	1	4	5	1	1	3	5
**16**	191	3	4	5	1	2	4	3	1	1	2
**16**	192	1	2	4	4	2	1	2	1	5	3
**17**	193	4	3	3	5	3	4	5	5	3	3
**17**	194	1	3	5	1	1	1	1	4	1	4
**17**	195	4	2	5	4	5	2	5	5	5	5
**17**	196	4	4	2	3	2	3	2	2	3	5
**17**	197	3	4	3	3	1	2	2	3	3	3
**17**	198	2	4	1	4	1	4	1	1	5	1
**17**	199	2	3	3	3	1	2	3	2	2	3
**17**	200	3	2	5	2	2	3	1	5	1	3
**17**	201	5	5	4	4	1	5	4	4	3	5
**17**	202	1	3	2	1	4	1	3	4	2	3
**17**	203	5	3	4	1	2	5	1	3	2	2
**17**	204	2	2	1	5	2	2	3	1	2	4
**18**	205	4	5	3	5	2	5	5	3	2	3
**18**	206	3	4	5	4	5	4	4	4	2	5
**18**	207	5	4	1	4	4	5	5	5	3	4
**18**	208	5	1	1	1	2	4	4	1	1	3
**18**	209	1	1	3	5	5	2	1	1	5	4
**18**	210	1	2	2	5	1	1	4	2	3	2
**18**	211	2	2	3	1	1	1	3	2	1	1
**18**	212	1	5	3	4	4	2	5	1	4	5
**18**	213	3	2	2	3	1	3	1	5	2	1
**18**	214	1	4	5	5	5	4	1	5	4	5
**18**	215	3	5	2	1	4	3	3	4	3	4
**18**	216	3	1	4	4	2	2	3	3	4	2
**19**	217	2	5	5	5	1	3	5	2	5	5
**19**	218	5	2	5	5	2	5	4	5	2	4
**19**	219	1	5	2	3	1	1	4	1	3	2
**19**	220	4	3	4	1	3	4	1	3	3	3
**19**	221	5	2	2	4	3	5	1	2	5	1
**19**	222	1	2	4	3	4	3	2	5	1	4
**19**	223	5	1	1	3	3	4	2	1	3	4
**19**	224	4	3	2	2	5	3	3	2	3	4
**19**	225	2	4	2	5	5	5	3	3	5	5
**19**	226	1	1	5	3	2	2	1	3	4	2
**19**	227	5	2	4	1	1	4	3	4	1	3
**19**	228	3	5	4	4	2	5	3	2	4	2
**20**	229	1	4	3	2	1	3	3	2	2	1
**20**	230	1	5	4	1	5	5	5	5	1	2
**20**	231	5	4	4	4	5	4	4	4	5	4
**20**	232	1	2	1	1	4	2	2	1	3	3
**20**	233	2	2	3	5	3	4	5	3	3	3
**20**	234	2	1	5	2	5	2	4	2	1	5
**20**	235	2	5	1	3	4	1	5	1	5	3
**20**	236	2	1	4	4	1	2	1	2	2	5
**20**	237	4	2	1	2	2	3	3	3	2	2
**20**	238	3	2	1	3	1	2	5	1	1	1
**20**	239	5	4	4	2	3	1	4	5	4	3
**20**	240	4	4	5	4	1	4	5	3	4	2
